# Relationship between mortality and health care expenditure: Sustainable assessment of health care system

**DOI:** 10.1371/journal.pone.0247413

**Published:** 2021-02-24

**Authors:** Phebe Asantewaa Owusu, Samuel Asumadu Sarkodie, Pål Andreas Pedersen

**Affiliations:** Nord University Business School (HHN), Bodø, Norway; University of Western Australia, AUSTRALIA

## Abstract

Infant and maternal mortality are important indicators for assessing the quality of healthcare systems. The World Health Organization underscores the importance of proper health care system in reducing preventable mortality through early intervention. Early intervention includes availability, accessibility and affordability of health care systems for children and mothers. While there are several studies that assess the immediate and underlying drivers of child mortality, literature on the role of policy measures are limited and inconsistent. Thus, robust empirical analysis of the determinants of maternal and infant mortality remains inconclusive in the era of achieving the Sustainable Development Goals (SDG). Here, we examined the influence of health expenditure on infant and maternal deaths for the period 2000–2015 across 177 countries. Using panel Quantile Regression with bootstrapping, this study accounted for the 2007–2008 financial crisis in an empirical relationship between health outcome and health expenditure. We found a negative effect of health expenditure on mortality across all percentiles. Infant mortality rate declines between 0.19% - 1.45% while maternal mortality rate declines ranging from 0.09% - 1.91%. To attain the goal of ensuring healthy lives and wellbeing of all people (SDG 3), this study infers that health expenditure potentially reduces maternal and infant mortality across lower and middle income countries. We highlight the need for an enhanced health care expenditure, especially in developing countries to curb the levels of infant and maternal deaths.

## Introduction

The impact of the health care system on human life (health outcome) has over the years triggered public health concern among policymakers to find a lasting solution (sustainable options). Thus, in the year 2015, the sustainable development goals (SDGs) were developed. The goal 3 of the SDGs, seeks to promote ‘good health and wellbeing for all and at every stage (from conception to old age) in one’s lifetime’ [1]. As part of achieving SDG 3 and universal health coverage, there is a need for good health care systems to be in place. The health care system is made up of various sectors or branches of which cost or financing cannot be left out as it is one of the objectives of SDG 3 (strengthening health financing).

In assessing the health status of any given country, maternal and infant mortality rate indicators of avoidable or treatable conditions play a vital role in assessing population health, quality of care, poverty, and socioeconomic status, among others. Approximately 303 thousand women died globally in 2015 owing to pregnancy and childbirth causes with the majority of deaths recorded in low- and middle-income nations. In 2016, 2.6 million deaths of new-borns were recorded [2].

Health status in general, as well as, the mortality rate at various stages of life in developed, developing, and least developed countries vary based on several factors. The government/health insurance/private funds (out-of-pocket, NGOs, private corporations) spending in health (health expenditures) are few of the factors that mortality rate varies greatly from country to country [3]. The mortality rates and health spending to some extent differ from state to state within countries.

Considering this, the study seeks to examine the overarching effect of health care expenditure on mortality (maternal and infant). Maternal and infant mortality are considered because, majorly, they are vulnerable in the society, and governments spending on health are mostly not categorized based on the type of care (primary, secondary, and tertiary) provided.

The relationship between health outcome (mortality) and health expenditure has been studied over the years either for an individual (time series) or a group of countries (such as OECD, sub-Saharan Africa). However, these studies have varying results with different data sets and methods used especially for maternal mortality ratio [4–10]. As pointed out in a recent study [9], an investigation of this kind requires accounting for “unobserved heterogeneity”, a challenge that most existing literature fails to address. As a contribution to health economics literature, this study employs a quantile estimation method with a bootstrapping technique that controls for heterogeneous parameters across countries and quantiles (0.05,…, 0.95). The advantage of this model estimation is that the conditional distribution of the nexus between mortality and expenditure can be estimated in detail across different quantiles (0.05,…, 0.95) ─ a situation that differs in extant literature. This characteristic of the panel data method is useful in global studies that consider diverse income groups and requires policy formulation for different scenarios ─ a conceptual tool that is required in this study.

Our study demonstrated that improving healthcare expenditure has greater impact in reducing infant and maternal mortality in developing countries compared to high-income countries. This implies that policies that make healthcare availability, accessibility and affordability in low-income countries, have mitigating effects on infant and maternal mortality. Thus, private and governmental bodies can invest and ensure appropriate allocation of health system-based monetary resources to achieve desirable health outcomes (reduced morbidity and mortality).

### Theoretical background of the study

Infant and maternal health outcomes are measures that gained a lot of attention in the early 90s in both developed and developing economies. To improve the health outcomes of mothers and children under age 5, the Millennium Development Goals (MDGs) 4 and 5 were set by United Nations (UN) member states in 2000 to achieve a reduction in child deaths and improve maternal health by 2015 [11]. At the end of the period, MDGs 4 and 5 were achieved to an extent in developed nations but was limited in developing and least-developed nations among the UN member states. Thus, the global infant mortality rate from 1990 (64.7 deaths per 1,000 live births) dropped considerably over the years by 54.6% (29.4 deaths per 1,000 live births in 2017) but its impact still lingers in developing countries. The annual maximum and minimum IMR for the study period is 142 and 1.7 deaths per 1,000 live births, respectively. MMR from 1990 (385 deaths per 100,000 live births) to 2015 (216 deaths per 100,000 live births) have been decreasing but at a slower pace compared to IMR in terms of the global average. MMR from 2000–2015 recorded as high as 2,650 deaths and as low as 3 deaths per 100,000 live births.

To resolve this in developing and underdeveloped nations, there is a school of thought that increasing health spending can reduce infant and maternal deaths while impacting universal health coverage. But, other contributing factors aside health spending play a critical role ─ of which, there exist several studies with varying results [4].

To better understand the situation at hand, a review of existing published literature on the topic is explored. The results of the nexus between health spending and mortality (under-5 and infant) using Ordinary Least Squares regression among other methods found health spending as an insignificant determinate of mortality [5]. A similar study examined how state health expenditure affects infant mortality among states in India for the period 1961 to 1999 using probit static model and distributed lag model [6]. The results show that government spending did appear not to save lives in India, but found otherwise for rural households sample by allowing lagged effects. In research on health expenditure and health outcomes for the period 1995–2005 for OECD countries, the findings showed a significant impact of health spending reducing infant mortality, increasing life expectancy, and increasing potential life years lost, but not valid for maternal mortality [7]. A study for seven (7) countries in the sub-Saharan region of Africa revealed that there exists a nexus between gender inequality, health expenditure, and maternal mortality for the study period [8]. The relationship between health expenditure and mortality (infant, under-5, and maternal) for the period 1995–2014 showed a significant relationship between health expenditure and child mortality [9]. However, an insignificant relationship was found between health expenditure and maternal mortality. In Mexico, a study for the period 2000–2015 was investigated between government health expenditure maternal health, and maternal mortality (focus on Mexican poor population). The findings showed the ineffectiveness of government health expenditure in reducing maternal mortality [10].

Disruptions in financial markets hamper economic development, thus, affecting livelihood and welfare (declines household income and expenditure). A study showed that the economic crisis of Peru (started in 1988) impacted infant mortality by recording more deaths than expected in the absence of the crisis [12]. Therefore, the global financial crisis (FC) affects health expenditure—as a share of economic growth, thus, justifies the controlling of the global FC period 2007–2008 in this study for the study period (2000–2015).

## Materials and methods

### Data

The data series examined in this analysis are annual data series of Infant Mortality rate (IMR, per 1,000 live births), Maternal mortality ratio (MMR, modelled estimate, per 100,000 live births), and Current health expenditure (CHE, % of GDP). We selected 177 countries (n = 177, see S1 Appendix in S1 File) from the seven continents (South America, North America, Europe, Antarctica, Africa, Asia, and Australia) of the world due to data limitation (availability and missing data). The period for the analysis spans from 2000 to 2015 (T = 16 periods). Data were obtained from the World development indicators [13]. S2 Appendix in S1 File illustrates the descriptive statistics of data variables for its raw and logarithmic transformed state. Current health expenditure is defined as the total spending on health care ‘goods’ and ‘services’ “*including personal health care [curative care*, *rehabilitative care*, *long-term care*, *ancillary services*, *and medical goods] and collective services [prevention and public services as well as health administration]*” [3]. This CHE spending does not factor in costs from investments (machinery, stocks of vaccines for emergency or outbreaks, information technologies, and buildings). The world CHE in 2016 was 10.02% of GDP. A [14] statistical examination of the data shows that the mean CHE is 6.09% of GDP while the maximum and minimum are 20.41% and 1.03% of GDP, respectively [13]. This provides evidence of varying health expenditure across countries. The selection and inclusion of health expenditure, as a proxy variable for enhanced health care delivery stem from the Sustainable Development Goal (SDG) 3 of ensuring and promoting a healthy lifestyle and wellbeing. Health expenditure plays a vital role in universal health coverage, and accessibility to quality and effective health care services [14].

Infant mortality rate (infants < age 1) and maternal mortality ratio ─ as the two (2) measures for health outcomes are considered as the core indicators of assessing health care systems. Infants and women are the most vulnerable in society, hence, infant and maternal mortality rates are thoughtful indicators when measuring the overall socioeconomic level of a country [15].

### Model estimation

Various models have been employed in the study of panel empirical analysis and likewise many have been used to estimate the relationship between health outcome and health expenditure. The adoption of panel modelling stems from the utilization of cross-country data across time-frequency. Our study followed the standard pathway for model estimation using econometric methods. We first examined the descriptive statistics of the data series and examined the geographical distribution using spatial maps in the form of choropleth. Second, we tested for panel unit roots to investigate the stationarity of the variables. Third, we tested for panel cointegration among the variables with modern techniques like Westerlund, Pedroni & Kao after meeting the preconditions of the panel unit root tests. Fourth, to avoid spurious regression in the model, the study proceeded to test for the presence of heterogeneity using a modified Wald test for groupwise heteroskedasticity in a fixed-effect regression model. Because countries are diversified and are not equal in terms of demographic characteristics, medical technology, burden of disease, and health infrastructure, among others, we utilized econometric methods that account for such heterogeneities, country-specifics, and unobserved common factors. Heterogeneous effects of health care expenditure and health outcomes are reported to affect future convergence towards sustainable health [16]. Hence, the utilization of robust empirical techniques is essential to the consistency and robustness of the empirical modeling. The fixed-effect regression model can be expressed as a linear specification presentation in Eq (1):

$${ln\;(IMR}_{i,t})|{ln\;(MMR}_{i,t})=\alpha +\delta_{i}+\beta *\;{lnCHE}_{i,t}+\varepsilon_{i,t}$$ (1)

Where *ln(IMR)* and *ln(MMR)* represent the logarithmic transformation of infant and maternal mortality, *δ_i_* denotes the country-specific fixed effects, *α, δ_i_*, and *β* are the parameter to be estimated, *lnCHE* is health expenditure and *ε_i,t_* is the i.i.d error term across countries *i* in time *t*. The output of the estimated parameters is diagnosed using the modified Wald test to ascertain homoskedastic deviation using the outputted Chi-square statistic.

From the fixed-effects model estimation, the study proceeded to apply the Panel Quantile Regression (QuanR) estimator with bootstrapping technique for empirical analysis, first for the whole study period (2000–2015), and secondly, considering the financial crisis (FC) period by estimating pre (2000–2006), during (2007–2008) and post (2009–2015) FC. To test the quality of the regression estimate, the empirical analysis produces a goodness-of-fit measure (pseudo-R-squared). The following are some of the advantages of QuanR as compared with standard linear regression (SLR) models [17]:

- QuanR estimates the relationship between the dependent and independent(s) variable(s) considering the ‘conditional distribution’ while SLR considers the ‘conditional mean analysis’.
- QuanR has a unique median regression (also known as the least absolute deviations, LAD estimator) that is additionally robust to outliers and errors that are non-normal when compared to SLR (least square estimates). This makes QuanR more efficient.
- In QuanR analysis, the variable(s) state remains unchanged when logarithmic transformations are used, easing results interpretation.

The panel QuanR (Q) for this study follows a *modified version* of the estimator introduced by Koenker and D’Orey [18]. Eq (2) shows the linear quantile specification, where *β*(*τ*) signifies the vector of coefficients associated with the *τ*–th quantile

τ=0.05,0.1,……,0.9,0.95

For brevity, the modelled equations for the analysis are as follows:

$${Q_{lnIMR}}_{i,t}[\tau |{lnCHE}_{i,t,}\beta (\tau )]=\alpha_{0,\tau }+\beta_{1,\tau }{lnCHE}_{i,t}\;i=1\ldots 177\;t=T$$ (2A)

$${Q_{lnMMR}}_{i,t}[\tau |{lnCHE}_{i,t,}\beta (\tau )]=\alpha_{0,\tau }+\beta_{1,\tau }{lnCHE}_{i,t}$$ (2B)

## Results and discussion

### Results

**Fig 1:**
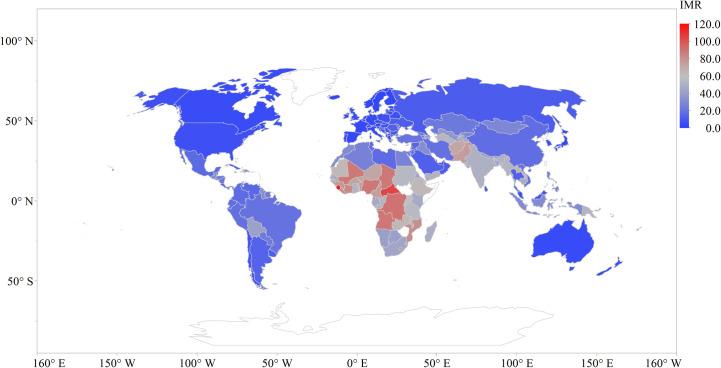
Infant mortality rate distribution across the globe.

**Fig 2:**
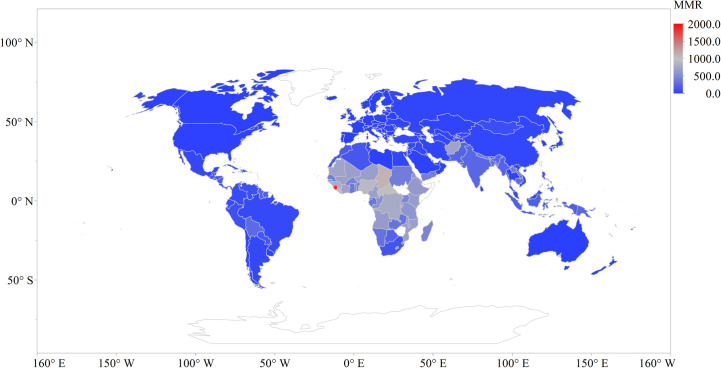
Maternal mortality rate distribution across the globe.

**Fig 3:**
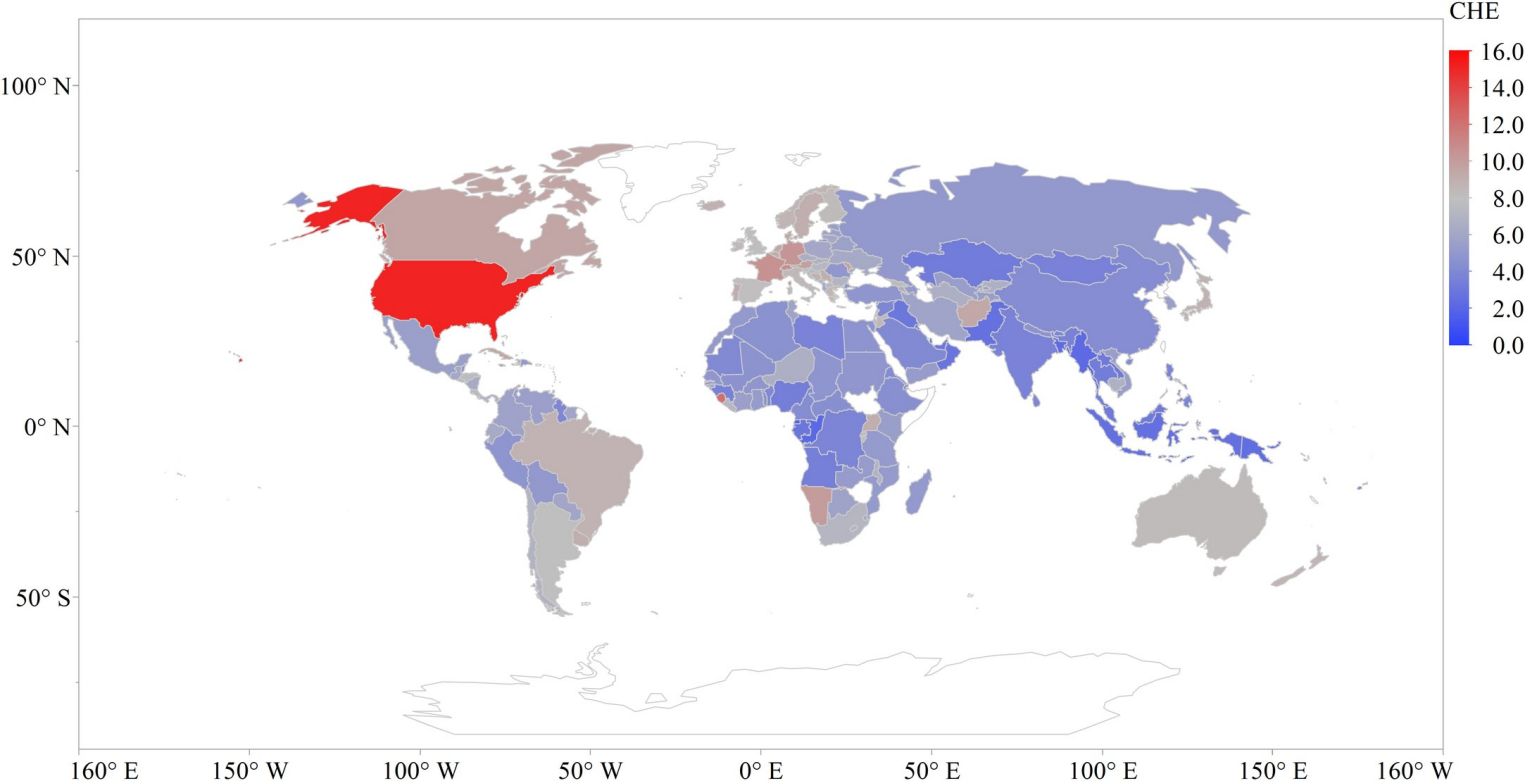
Health care expenditure across the globe.

Figs 1–3 show the average raw data distribution of IMR, MMR, and CHE across the globe for the period of study. From the IMR choropleth map (Fig 1) it can be observed that the deep blue coloured areas are countries with very low infant mortality rates. The countries with lower IMR can be spotted in Southern America, North America, Europe, Antarctica, Asia (except Pakistan and Afghanistan), and Australia. Sub-Saharan Africa records the highest IMR with Democratic Republic of Congo (90.6 deaths per 1000 live births), Central African Republic (104 deaths per 1000 live births), and Sierra Leone (117 deaths per 1000 live births) as the top three records for the study period.

The MMR choropleth map (Fig 2) shows that Sierra Leone (1874), Chad (1103), South Sudan (1007), Central African Republic (1006), and Liberia (952) shows the top five (5) deaths per 100,000 live births. These countries are all in the Sub-Saharan African region with Sierra Leone recording the highest average number of maternal deaths for the study period. The lowest number of maternal deaths are in the other continents except for Afghanistan (718 deaths per 100,000 live births).

The United States recorded the highest spending (Fig 3) on health (15.2%) for the study period followed by Micronesia (10.9%), Switzerland (10.74%), Kiribati (10.72%), and Germany (10.5%). In the sub-Saharan African region Sierra Leone (12.2%), Namibia (10.1%), and Uganda (9.1%) were recorded as the top three countries with the averaged maximum spending on health for the study period.

Prior to modelling the nexus between mortality and health expenditure, we conducted a series of tests including panel unit root and cointegration tests. The variability of mortality and expenditure (a share of economic growth) makes the data series prone to fluctuations based on either a common factor or unobserved factors. S3 Appendix in S1 File reveals that some series in the panel is stationary at first difference, thus, rejecting the null hypothesis that the panel contains a unit root. The concept of cointegration is only applicable if the variables are integrated of order one, I(1)—as revealed in this study. After testing for panel unit roots, the study proceeded to examine the panel cointegration of the models using Westerlund, Pedroni, and Kao tests. The results of the panel cointegration in Table 1 shows a rejection of the null hypothesis of no cointegration at 1% and 5% significant level. This confirms the existence of a level relationship between health spending (CHE) and health outcomes [mortality (IMR and MMR)].

**Table 1:** Panel cointegration using Westerlund, Pedroni & Kao tests.

IMR~f(CHE)			MMR~f(CHE)		
*Westerlund*	Statistic	p-value	*Westerlund*	Statistic	p-value
Variance ratio	14.0557	<0.0001	Variance ratio	10.6451	<0.0001
*Pedroni*			*Pedroni*		
Modified Phillips-Perron t	9.6750	<0.0001	Modified Phillips-Perron t	6.6939	<0.0001
Phillips-Perron t	-3.0517	0.0011	Phillips-Perron t	-6.2586	<0.0001
Augmented Dickey-Fuller t	2.1918	0.0142	Augmented Dickey-Fuller t	-2.5276	0.0057
*Kao*			*Kao*		
Modified Dickey-Fuller t	11.5504	<0.0001	Modified Dickey-Fuller t	3.7931	0.0001
Dickey-Fuller t	9.3779	<0.0001	Dickey-Fuller t	-5.0551	<0.0001
Augmented Dickey-Fuller t	15.6777	<0.0001	Augmented Dickey-Fuller t	1.9953	0.0230
Unadjusted modified Dickey	7.6277	<0.0001	Unadjusted modified Dickey	6.7373	<0.0001
Unadjusted Dickey-Fuller t	1.6754	0.0469	Unadjusted Dickey-Fuller t	-2.4979	0.0062

After confirming cointegration among the variables, the study proceeded to test for heterogeneity using a modified Wald test for groupwise heteroskedasticity in a fixed-effect regression model. Heterogeneity in standard panel data models leads to nuisance and spuriousity in estimated parameters if not controlled [19]. Results of the fixed-effect regression in S4 Appendix in S1 File show that an increase in health expenditure declines both maternal and infant mortality. However, the distributed Chi-squared (*χ*^2^) of the modified Wald test statistic shows that the null hypothesis of homoskedasticity is rejected at 1% significance level—confirming the presence of heterogeneity in the panel-based estimated model. Thus, a re-estimation of the panel regression model using econometric techniques that account for heterogeneity was essential. Similar studies [9, 20] have thus far applied standard econometric techniques based on mean regression—with statistical inferences that can miss useful policy information due to cross-sectional dependence and heterogeneity. In contrast, this study applied a panel quantile regression technique that accounts for unobserved heterogeneity and the conditional distribution of mortality across quantiles in 177 countries.

The estimation technique employed in this study is based on panel quantile regression with country-specific fixed effects. Contrary to traditional estimation methods, the panel quantile regression captures country-specific unobserved heterogeneity uncontrolled for by other covariates in the estimated model [21]. Hence, the effect of health expenditure on mortality rate was properly estimated—since idiosyncratic error due to the departure from country-specific idiosyncratic levels and heterogeneity bias were controlled. Tables 2 and 3 show the results of the estimated parameters that examine the heterogeneous distributional relationship between mortality rates and health expenditure. Table 2 shows that the estimated coefficients from 5^th^ to 95^th^ percentiles are negative and statistically significant at 1% level; however, the magnitude of the coefficient increases after 40^th^ percentile. The empirical results show that a 1% increase in health expenditure decreases infant mortality by ~0.19 to ~1.45%. Thus, countries with low infant mortality rate are more responsive to health expenditure compared to countries with high infant mortality rate. Table 3 reveals negative and statistically significant coefficients (*p<0*.*01*) from 0.05 quantile to 0.95 quantile—in the nexus between maternal mortality rate and heterogeneous health expenditure. In the same manner, a 1% increase in health expenditure decreases maternal mortality rate by ~0.09 to ~1.91%. In contrast to countries with high maternal mortality rate, the mitigating effect of health expenditure on maternal mortality rate is highly visible in countries with low maternal mortality.

**Table 2:** Infant mortality-health expenditure nexus.

lnIMR	lnCHE, coef.	C	R^2^	S.E	QD	SP	P-value	Q.LR
**0.05 q**	-0.8958* [0.0463]	2.9193* [0.0834]	0.0938	1.8461	1.1632	5.1428	<0.0001	215.3996
**0.1 q**	-0.8535* [0.0428]	3.0579* [0.0762]	0.1153	1.6724	1.3350	3.9088	<0.0001	339.1508
**0.2 q**	-1.2456* [0.0406]	4.1564* [0.0778]	0.1441	1.3639	1.8245	3.0642	<0.0001	530.1653
**0.3 q**	-1.4519* [0.0335]	4.8036* [0.0557]	0.1393	1.2019	2.2721	2.6163	<0.0001	580.5745
**0.4 q**	-1.3386* [0.0505]	4.8898* [0.0789]	0.1098	1.0737	2.6946	2.6929	<0.0001	428.1503
**0.5 q**	-1.0839* [0.0418]	4.7632* [0.0648]	0.0887	1.0039	2.9806	2.7313	<0.0001	334.1748
**0.6 q**	-0.8879* [0.0409]	4.7385* [0.0644]	0.0679	1.0396	3.3393	3.0897	<0.0001	223.9271
**0.7 q**	-0.7178* [0.0866]	4.8002* [0.1335]	0.0380	1.1885	3.7257	3.8243	<0.0001	99.7327
**0.8 q**	-0.3812* [0.0521]	4.6298* [0.0797]	0.0197	1.4643	4.0271	2.9460	<0.0001	65.7563
**0.9 q**	-0.2526* [0.0435]	4.6930* [0.0727]	0.0112	1.6874	4.2918	3.0925	<0.0001	35.3947
**0.95 q**	-0.1885* [0.0391]	4.7672* [0.0691]	0.0089	1.8408	4.4578	4.1812	<0.0001	21.1321

Notes: lnCHE, shows the marginal effect, q means Quantile, R^2^ represents Pseudo R-squared, S.E is the standard error of the regression, QD is the Quantile dependent var, SP denotes Sparsity, Q.LR means Quasi-LR statistic and P-value is the probability of the Quasi-LR statistic.

* represents 1% significance level (p < 0.01), and the *Standard error* in parentheses [..].

**Table 3:** Maternal mortality-health expenditure nexus.

lnMMR	lnCHE, Coef.	C	R^2^	S.E	QD	SP	P-value	Q.LR
**0.05 q**	-0.8687* [0.1046]	3.3624* [0.2008]	0.0485	2.8278	1.6094	8.1874	<0.0001	98.9611
**0.1 q**	-1.0772* [0.0764]	4.0381* [0.1403]	0.0682	2.5669	1.9459	5.6789	<0.0001	195.6367
**0.2 q**	-1.5281* [0.0809]	5.4442* [0.1581]	0.0937	2.1081	2.4849	4.6194	<0.0001	327.7249
**0.3 q**	-1.8500* [0.0638]	6.3825* [0.1183]	0.1095	1.8910	3.2189	3.7656	<0.0001	469.1974
**0.4 q**	-1.9095* [0.0639]	6.8720* [0.1060]	0.0896	1.7194	3.6889	3.9471	<0.0001	361.5214
**0.5 q**	-1.5591* [0.0721]	6.7394* [0.1067]	0.0716	1.5861	4.1271	4.5212	<0.0001	252.7391
**0.6 q**	-1.2956* [0.0808]	6.7822* [0.1317]	0.0622	1.6068	4.7005	4.9889	<0.0001	202.0883
**0.7 q**	-1.0002* [0.1399]	6.9252* [0.2367]	0.0383	1.8577	5.3845	6.7938	<0.0001	91.8318
**0.8 q**	-0.5353* [0.0936]	6.8438* [0.1345]	0.0108	2.3369	6.0064	5.8254	<0.0001	29.8214
**0.9 q**	-0.2841* [0.0703]	6.9598* [0.1178]	0.0041	2.7759	6.4831	4.8821	<0.0001	13.1772
**0.95 q**	-0.0896* [0.0505]	6.8195* [0.0827]	0.0013	2.9484	6.6758	5.8014	<0.0001	3.4731

Notes: lnCHE, shows the marginal effect, q means Quantile, R^2^ represents Pseudo R-squared, S.E is the standard error of the regression, QD is the Quantile dependent var, SP denotes Sparsity, Q.LR means Quasi-LR statistic and P-value is the probability of the Quasi-LR statistic.

* represent 1% significance level (p < 0.01), and the Standard error in parentheses [..].

### Model validation

Model validation is essential to investigate residual independence, thus, critical for robust results with unbiased statistical inferences. The estimated panel quantile regression was validated using diagnostic tests, quantile process coefficient test, slope equality test, and a re-estimation of the models by controlling for global financial crisis shocks. Our panel quantile regression was estimated using the Markov-Chain Marginal bootstrap method with 10,000 replications for coefficient covariance estimation, and Epanechnikov kernel residual with Hall-Sheather bandwidth method for sparsity estimation.

Tables 2 and 3 reveal that the sparsity-based Quasi-LR statistic in almost all the quantiles has a statistically significant probability at 1% level. In Table 4, we examined the quantile slope equality using Wald test as a robust technique for testing heteroskedasticity. The results of the conventional test levels for 5^th^-95^th^ percentiles are significant at 1% level, hence, the conditional quantiles are non-identical and the estimated coefficients in both models differ across quantiles.

**Table 4:** Quantile slope equality test summary.

Wald Test	Chi-Sq. Statistic^a^	Prob. ^a^	Chi-Sq. Statistic^b^	Prob. ^b^
***5***^***th***^ ***percentile***	833.6807	<0.0001	503.9763	<0.0001
***10***^***th***^ ***percentile***	833.3980	<0.0001	495.5384	<0.0001
***20***^***th***^ ***percentile***	833.3980	<0.0001	495.7348	<0.0001
***30***^***th***^ ***percentile***	828.4113	<0.0001	495.5384	<0.0001
***40***^***th***^ ***percentile***	833.3980	<0.0001	495.7348	<0.0001
***50***^***th***^ ***percentile***	828.4113	<0.0001	495.7370	<0.0001
***60***^***th***^ ***percentile***	833.3980	<0.0001	495.7348	<0.0001
***70***^***th***^ ***percentile***	833.3980	<0.0001	495.9524	<0.0001
***80***^***th***^ ***percentile***	829.4048	<0.0001	496.1406	<0.0001
***90***^***th***^ ***percentile***	833.3980	<0.0001	495.9313	<0.0001
***95***^***th***^ ***percentile***	941.9810	<0.0001	713.4720	<0.0001

Notes

^**a**^ denotes model specification lnIMR~f(lnCHE) while

^**b**^ denotes lnMMR~f(lnCHE)

The re-estimation of the models by accounting for the financial crisis shocks—with results presented in Tables 5–7. For brevity, 5^th^, 50^th^, and 95^th^ percentiles—denoting low, medium, and high mortality (infant and maternal) rates were estimated. In the pre-global financial crisis (2000–2006) presented in Table 5, the mitigating effect of heterogeneous health expenditure is high in countries with medium-infant mortality rate, medium in countries with low-infant mortality rate, and low in countries with high-infant mortality rate. The same trend occurs with maternal mortality-health expenditure nexus, except that, the impact of health expenditure in countries with high-infant mortality rate turns statistically insignificant—due to identical conditional quantiles and equal estimated coefficients across quantiles (see p-value of Quasi-LR statistic at 95^th^ percentile in Table 5).

**Table 5:** Mortality-health expenditure nexus from 2000–2006 (pre-global financial crisis).

	lnCHE, Coef.	C	R^2^	S.E	QD	SP	P-value	Q.LR
lnIMR								
0.05 q	-0.8296* [0.0532]	2.9246* [0.0907]	0.0916	1.8581	1.3350	5.1107	<0.0001	90.4516
Median q	-1.0576* [0.0637]	4.8369* [0.0886]	0.0670	1.0075	3.1485	2.9565	<0.0001	101.0224
0.95 q	-0.1794* [0.0410]	4.8447* [0.0658]	0.0116	1.7863	4.5716	3.6503	0.0003	13.1936
lnMMR								
0.05 q	-1.1247* [0.1705]	3.9330* [0.3155]	0.0509	2.7927	1.7918	8.0718	<0.0001	45.8481
Median q	-1.6587* [0.1150]	6.9313* [0.1886]	0.0750	1.5954	4.2195	4.2434	<0.0001	122.9733
0.95 q	-0.1105 [0.0955]	6.9224* [0.1523]	0.0007	2.8993	6.7662	5.6710	0.3409	0.9071

Notes: lnCHE, shows the marginal effect, q means Quantile, R^2^ represents Pseudo R-squared, S.E is the standard error of the regression, QD is the Quantile dependent var, SP denotes Sparsity, Q.LR means Quasi-LR statistic and P-value is the probability of the Quasi-LR statistic.

* represents 1% significance level (p < 0.01), and the Standard error in parentheses [..].

**Table 6:** Mortality-health expenditure nexus from 2007–2008 (during the global financial crisis).

	lnCHE, Coef.	C	R^2^	S.E	QD	SP	P-value	Q.LR
lnIMR								
0.05 q	-0.8232* [0.1688]	2.7234* [0.3103]	0.0809	1.8960	1.1632	5.8691	<0.0001	20.0304
Median q	-1.0183* [0.1452]	4.6225* [0.2313]	0.0674	1.0224	2.9339	3.0025	<0.0001	28.6970
0.95 q	-0.0878 [0.0669]	4.5723 [0.1165]	0.0070	1.8356	4.4140	4.4237	0.1704	1.8792
lnMMR								
0.05 q	-0.6308* [0.2696]	2.8086* [0.5111]	0.0371	2.9516	1.6094	8.3313	0.0024	9.2444
Median q	-1.2204* [0.2522]	6.1326* [0.3992]	0.0385	1.6212	4.0775	5.0038	0.0001	15.4118
0.95 q	0.0809 [0.1545]	6.5077* [0.2677]	0.0005	2.9557	6.6134	6.9039	0.7051	0.1432

Notes: lnCHE, shows the marginal effect, q means Quantile, R^2^ represents Pseudo R-squared, S.E is the standard error of the regression, QD is the Quantile dependent var, SP denotes Sparsity, Q.LR means Quasi-LR statistic and P-value is the probability of the Quasi-LR statistic.

* represents 1% significance level (p < 0.01), and the *Standard error* in parentheses [..].

**Table 7:** Mortality-health expenditure nexus from 2009–2015 (post-global financial crisis).

	lnCHE, Coef.	C	R^2^	S.E	QD	SP	P-value	Q.LR
lnIMR								
0.05 q	-0.8404* [0.0620]	2.7094* [0.1205]	0.0917	1.8282	0.9555	5.5402	<0.0001	83.8396
Median q	-1.1533* [0.0715]	4.7872* [0.1056]	0.1089	0.9821	2.7973	2.7400	<0.0001	177.5534
0.95 q	-0.1523* [0.0549]	4.4985* [0.0912]	0.0088	1.7948	4.2781	3.8592	0.0018	9.7249
lnMMR								
0.05 q	-0.8592* [0.1315]	3.2725* [0.2583]	0.0496	2.8060	1.6094	7.8789	<0.0001	45.7502
Median q	-1.5718* [0.1257]	6.7475* [0.1651]	0.0773	1.5651	4.0254	5.0378	<0.0001	107.0256
0.95 q	-0.1015 [0.1030]	6.7037* [0.1716]	0.0005	2.9326	6.5681	6.5027	0.4532	0.5626

Notes: lnCHE, shows the marginal effect, q means Quantile, R^2^ represents Pseudo R-squared, S.E is the standard error of the regression, QD is the Quantile dependent var, SP denotes Sparsity, Q.LR means Quasi-LR statistic and P-value is the probability of the Quasi-LR statistic.

* represents 1% significance level (p < 0.01), and the Standard error in parentheses [..].

During the global financial crisis (2007–2008) presented in Table 6, the impact of health expenditure in reducing both infant and maternal mortality rate is high in medium-infant mortality rate countries and medium in low-infant mortality rate countries. However, due to identical conditional quantiles and homogeneous coefficients across quantiles in both models, the effect of health expenditure on mortality in high-mortality rate countries becomes insignificant (see p-value of Quasi-LR statistic at 95^th^ percentile in Table 6).

In the post-global financial crisis (2009–2015) presented in Table 7, health expenditure negates both infant and maternal mortality in medium mortality rate countries by 1.15% (infant) and 1.57% (maternal), low mortality rate countries by 0.84% (infant) and 0.86% (maternal), and countries with high-infant mortality rate by 0.15%. In contrast, the impact of health expenditure on maternal mortality in countries with high-maternal mortality rate turns statistically insignificant due to the equal coefficients across quantiles and identical conditional quantiles (see p-value of Quasi-LR statistic at 95^th^ percentile in Table 7).

To test the robustness of the estimated parameters, we employed quantile process coefficient test based on a graphical representation depicted in S5 Appendix in S1 File. A visual inspection of the corresponding figures reveal that the estimated coefficients are within the 95% confidence band, hence, confirms the robustness and stability of the estimated models.

## Discussion

As this study sought to add its contribution to the growing debate by studying CHE impact on IMR and MMR for the MDGs year-span (2000–2015) using QuanR as well as considering the Financial Crisis (FC) period of 2007–2008 [22].

Empirical analysis results show that the relationship between health expenditure and IMR and MMR are statistically significant with varying degrees of positive impact across the 11 quantiles for the first part of the analysis without considering FC periods. An increase in health expenditure by 1% shows a relatively greater reduction on infant deaths from the 20^th^ percentile to the median with a significant impact at the 30^th^ percentile by ~1.45%, while that of maternal deaths is observed between the 10^th^– 70^th^ percentile and majorly at the 40^th^ percentile by ~1.91%. Increasing health expenditure shows minimal impact at the upper end of the conditional distribution (80^th^– 95^th^ percentile). Results by Akinlo and Sulola [23] disagree with our outcome on health expenditure reducing infant mortality but the findings by Rana, Alam [9] and Nicholas, Edward [24] are consistent with ours. The empirical results of the nexus between maternal mortality and health expenditure by Nicholas, Edward [24] and Rana, Alam [9] are inconsistent with our outcome.

The results infer that although health expenditure increase reduces IMR and MMR for all economies under-study, countries at the verge of beginning their mid-income status or developing nations will benefit more in reducing death rate than countries categorised as least developed and developed economies. This can perhaps be explained by environmental and health awareness with increasing level of income that moderates the greater effect of healthcare expenditure on infant and maternal mortality rates across countries en route to middle-income. The findings can be attributed to the fact that in low-income economies, an increase in health expenditure mitigates mortality rates to an extent. Other factors such as delay in seeking proper medical attention, self-medication, among others (education, infrastructural problems, resource allocation, poor sanitation, health governance, etc.) cannot be overlooked as contributing factors. A study by Bhalotra [6] on health spending and infant mortality in India showed that health spending do not save infants from death, however, by considering certain specifications such as ‘mother’s age at birth’, ‘education’, etc. they found otherwise corroborating the point made earlier. Thus, the share of GDP for the health sector in developing countries should be enhanced due to its role in mitigating IMR and MMR. In high-income countries, other unaccounted factors in this study might exacerbate mortality rates, hence, further research will be worthwhile.

The 2007–2008 FC period showed no causal relationship between CHE and IMR and MMR for developed nations (95^th^ quantile). The results of least-developed (5^th^ quantile) and developing (50^th^ quantile) countries were similar to pre-, during, and post- global FC in reducing IMR and MMR. The FC period did have an effect on mortality (IMR and MMR), this effect was observed prominently for the median quantile of maternal mortality ratio since the marginal coefficient of lnCHE during the FC period (2007–2008) was less (~1.22%) compared to the pre-FC (~1.66%) and post-FC (~1.57) period.

**Fig 4:**
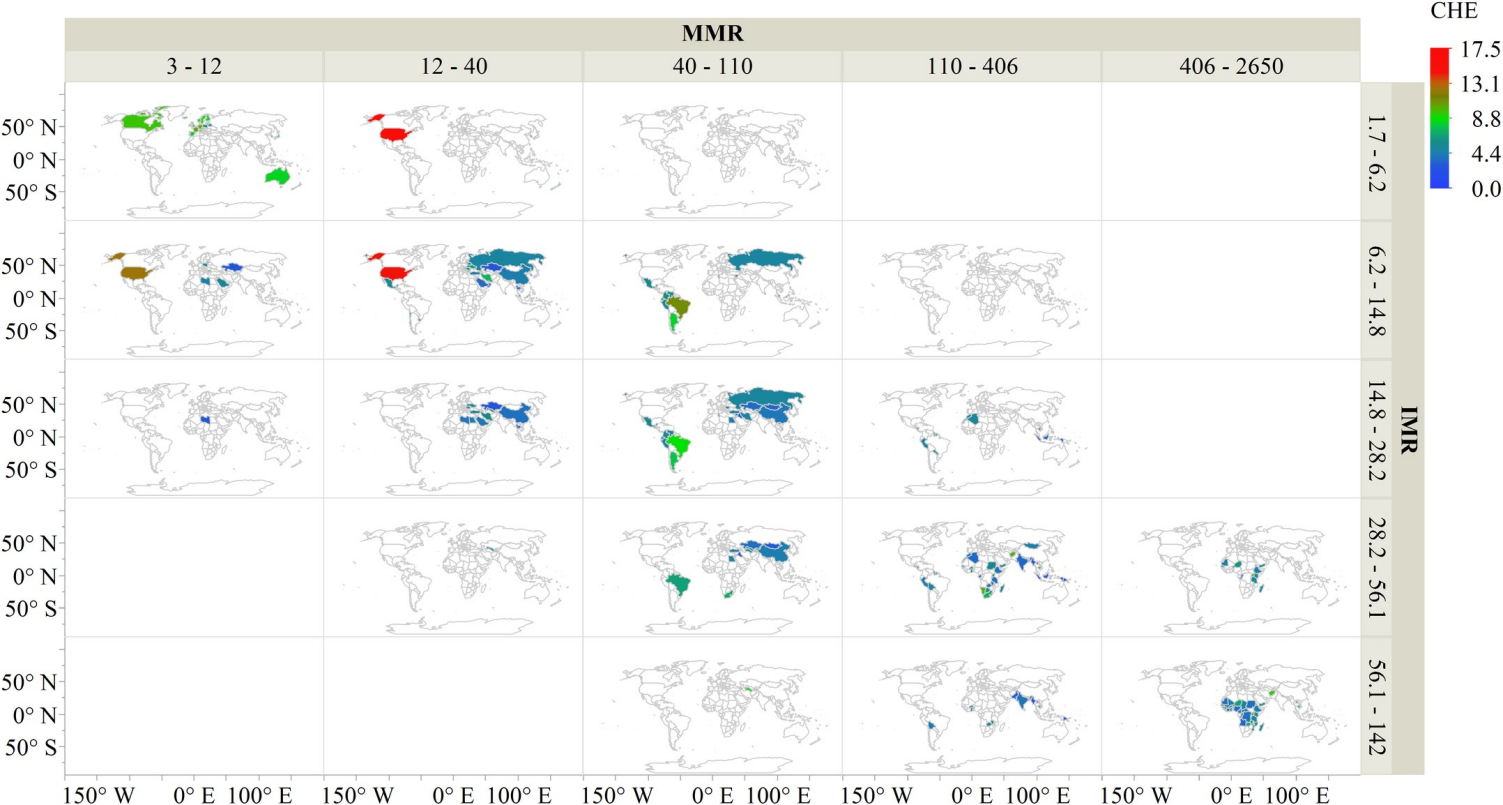
Infant mortality rate and maternal mortality ratio distribution across the globe.

In summary Fig 4 illustrates that in general, as health expenditure is increased, infant mortality and maternal mortality decreases. A visual inspection of Fig 4 and graphs in S5 Appendix in S1 File. shows that health expenditure can only mitigate mortality rates to an extent.

To have a lasting solution further studies should focus on the health care system as a whole and ways to minimize corrupt practices in least-developed and developing economies [25]. There is evidence of female education reducing infant mortality [4], hence, governments and stakeholders should put measures in-place to formally educate several women.

## Conclusion

The nexus between health expenditure, infant mortality rate, and maternal mortality ratio has been studied extensively over the years. In the extant literature, most of the analysis between health expenditure and health outcome employed models that consider the conditional mean analysis. In contrast, this paper employed the conditional distribution analysis (Quantile Regression) to study the effect of health expenditure on health outcomes for the period 2000–2015 for 177 countries. Secondly, the study considered the global financial crisis of 2007–2008. The results showed a significant positive relationship between health expenditure and infant mortality rate, and maternal mortality ratio for the study period across 11 quantiles ─ with the most impact observed in developing economies.

Considering the effect of the global financial crisis (2007–2008), health expenditure had a mitigating effect on infant and maternal mortality for least developed and developing economies contrary to developed countries. Multidimensional poverty is often high in developing countries, especially low-income economies, hence, limits access to affordable healthcare system. Thus, healthcare policies in developing countries that favour the poor contribute significantly in improve health and wellbeing. The removal of fiscal barriers to proper healthcare in poor countries is reported to reduce mortality while improving health outcomes. Factually, these inferences draw our attention that, increasing health care expenditure mitigates mortality to an extent. Therefore, as the study period showed that health expenditure does reduce mortality in the MDG’s era (2000–2015), there is no doubt that increasing health spending will play a vital role in reducing mortality and aid in achieving SDG 3.

This study confirmed the nexus between health expenditure, infant-, and maternal mortality ─ showing that an increase in health expenditure will decline maternal deaths contrary to other reported literature that found no significant causal relationship.

We further observe from the mapping that Senegal’s health expenditure spending is relatively high compared to other African countries in the sub-region, however, its mortality rates are also high. This could be attributed to misappropriation of funds or other underlying factors in the health care system including limited health facilities, lack of infrastructure, lack of qualified personnel, and lack of medication. This challenges cut across in several developing economies, hence, an indepth research into the specific cause(s) is warranted. Therefore, the institution of sustainable healthcare-related measures will further reduce infant and maternal deaths. Further research should focus on other measures that have a significant influence on health outcomes (mortality and morbidity).

## Supporting information

S1 File (PDF)
