# Supplementary material for: Relationship between mortality and health care expenditure: Sustainable assessment of health care system
Source: PLoS One. 2021 Feb 24;16(2):e0247413. doi: 10.1371/journal.pone.0247413 (PMC7904168; doi:10.1371/journal.pone.0247413)

APPENDICIES

S1 Appendix. Study Countries.

A

Afghanistan
Albania
Algeria
Angola
Argentina
Armenia
Australia
Austria
Azerbaijan

B

Bahrain
Bangladesh
Barbados
Belarus
Belgium
Belize
Benin
Bhutan
Bolivia
Bosnia and Herzegovina
Botswana
Brazil
Brunei
Bulgaria
Burkina Faso
Burundi

C

Cabo Verde
Cambodia
Cameroon
Canada
Central African Republic
Chad
Chile
China
Colombia
Comoros
Congo
Costa Rica
Côte d'Ivoire
Croatia
Cuba
Cyprus
Czech Republic

D

Democratic Republic Congo
Denmark
Djibouti
Dominican Republic

E

Ecuador
Egypt
El Salvador
Equatorial Guinea
Eritrea
Estonia
Eswatini
Ethiopia

F

Fiji
Finland
France

G

Gabon
Georgia
Germany
Ghana
Greece
Grenada
Guatemala
Guinea
Guinea-Bissau
Guyana

H

Haiti
Honduras
Hungary

I

Iceland
India
Indonesia
Iran
Iraq
Ireland
Israel
Italy

J

Jamaica
Japan
Jordan

K

Kazakhstan
Kenya
Kiribati
Korea
Kuwait
Kyrgyz Republic

L

Lao PDR
Latvia
Lebanon
Lesotho
Liberia
Libya
Lithuania
Luxembourg

M

Madagascar
Malawi
Malaysia
Maldives
Mali
Malta
Mauritania
Mauritius
Mexico
Micronesia
Moldova
Mongolia

Montenegro

Morocco

Mozambique

Myanmar

N

Namibia

Nepal

Netherlands

New Zealand

Nicaragua

Niger

Nigeria

North Macedonia

Norway

O

Oman

P

Pakistan

Panama

Papua New Guinea

Paraguay

Peru

Philippines

Poland

Portugal

Q

Qatar

R

Romania

Russia

Rwanda

S

Samoa

São Tomé and Principe

Saudi Arabia

Senegal

Serbia

Sierra Leone

Singapore

Slovak Republic

Slovenia

Solomon Islands

South Africa

Spain

Sri Lanka

St. Lucia

St. Vincent and the Grenadines

Sudan

Suriname

Sweden

Switzerland

Syrian Arab Republic

T

Tajikistan

Tanzania

Thailand

The Bahamas

The Gambia

Timor-Leste

Togo

Tonga

Trinidad and Tobago

Tunisia

Turkey

Turkmenistan

U

Uganda

Ukraine

United Arab Emirates

United Kingdom

United States

Uruguay

Uzbekistan

V

Vanuatu

Venezuela

Vietnam

Yemen

Z

Zambia

Note: Selection based on data availability and countries with no missing data.

S2 Appendix. Descriptive statistics of the 177 countries.

Statistics	IMR	lnIMR	CHE	lnCHE	MMR	lnMMR
**Mean**	30.6526	2.9308	6.0909	1.7184	206.9209	4.2167
**Median**	19.75	2.9832	5.7443	1.7482	62.00	4.1271
**Maximum**	142.00	4.9558	20.4146	3.0163	2650.00	7.8823
**Minimum**	1.70	0.5306	1.0250	0.0247	3.00	1.0986
**Std. Dev.**	27.8432	1.0755	2.4921	0.4351	291.7327	1.6521
**Skewness**	1.0802	-0.2317	0.7102	-0.4806	2.3117	-0.0076
**Kurtosis**	3.3592	1.9486	3.9102	3.1905	11.2755	1.8434
**Jarque-Bera**	563.184	154.996	334.145	112.747	10551.16	157.102
**Probability**	<0.0001	<0.0001	<0.0001	<0.0001	<0.0001	<0.0001

S3 Appendix. Panel unit root tests.

Variables	Level		First-difference	
	Fisher	IPS	Fisher	IPS
lnCHE	0.6707	-1.1008	-9.6011*	-21.6950*
lnMMR	1.7899	-	-29.7252*	-
lnIMR	6.3858	17.1666	-1.3830***	-1.7446**

Note: * , **, *** represent 1% , 5% and 10% significance level

S4 Appendix. Modified Wald test for groupwise heteroskedasticity in fixed-effect regression model

Results	lnIMR	lnMMR
lnCHE	-0.2973*	-0.1286*
	[0.0241]	[0.0238]
_cons	3.4417*	4.4377*
	[0.0416]	[0.0411]
sigma_u	1.0194	1.6281
sigma_e	0.1922	0.1945
rho	0.9657	0.9859
Obs	2,818	2,818
Groups	177	177
Diagnostics		
Prob > F	<0.0001*	<0.0001*
R^2	0.13	0.10
χ^2	2.1e+05	8.5e+05
Prob $>\chi^2$	<0.0001*	<0.0001*

Note: * , **, *** represent 1% , 5% and 10% significance level

S5 Appendix. Quantile Process Estimation.

5th mmr

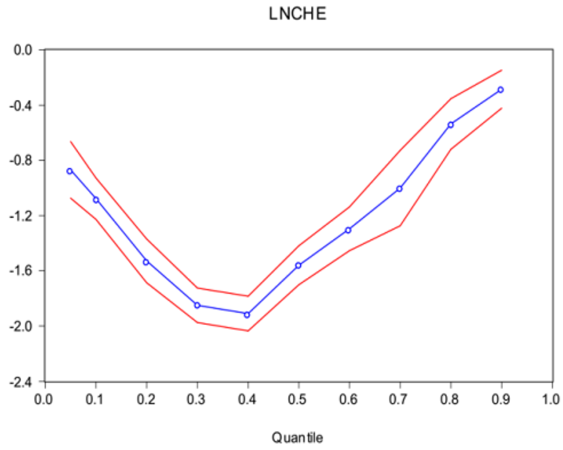

95th mmr

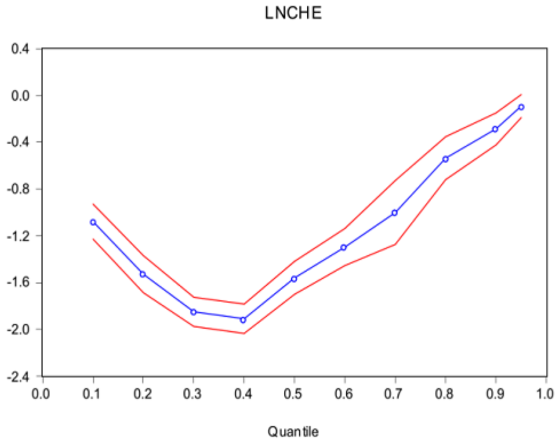

5th imr

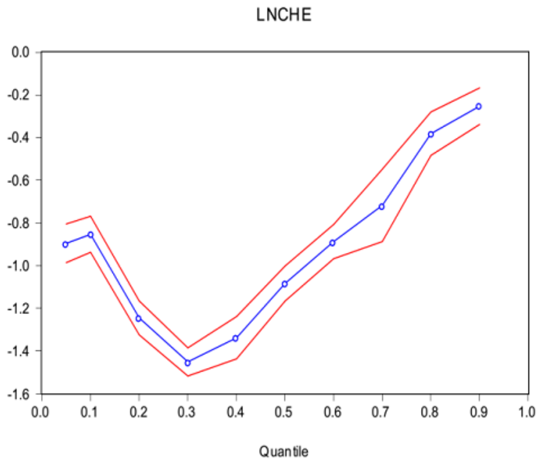

95th imr

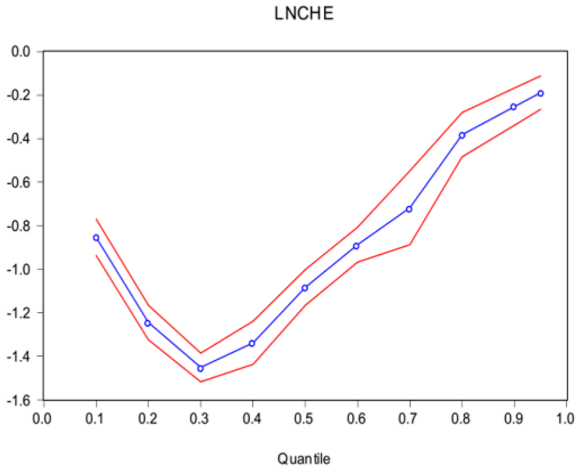

Supplement: S1 File — (PDF) [file pone.0247413.s001.pdf]
